# The Internet *versus* pediatricians as a source of infant teething information for parents in Turkey

**DOI:** 10.6061/clinics/2016(08)04

**Published:** 2016-08

**Authors:** Eda Haznedaroglu, Ali Mentes

**Affiliations:** Marmara University, Department of Pediatric Dentistry, Basibuyuk Campus, 9/3 34854 Basibuyuk, Maltepe/Istanbul/Turkey

**Keywords:** Infant, Teething, Website, Pediatrician

## Abstract

**OBJECTIVES:**

Parents are increasingly searching the Internet to gather information about their children’s health care. This study compared infant teething information obtained from publically employed pediatricians in Istanbul with that obtained from different Turkish websites (parenting, health, professional, news and commercial).

**METHODS:**

This study had two parts. The first part used a descriptive design, with two checklists to assess the quality and comprehensiveness of the teething-specific content on 62 parenting or health websites. The second part was a cross-sectional study of 75 pediatricians at public hospitals who completed a structured self-administered questionnaire.

**RESULTS:**

In total, 54 websites (87.1%) described infant teething as a normal developmental process. The lists that were found on the websites identified the most frequent signs of infant teething as fever and drooling/perioral rash. The most frequent management strategies were chewing non-chilled and chilled objects. For teething problems, some pediatricians recommended teething rings and oral benzocaine, while 23 pediatricians recommended nothing.

**CONCLUSIONS:**

Parents should be informed by health professionals, especially regarding specific treatment strategies.

## INTRODUCTION

Infant teething is a natural biological process that usually begins 4–10 months after birth and continues until the teeth move from their sites of development to their final functional positions in the oral cavity 1-2. Teething leads to local physiological transformations that may be inflammatory or irritative in nature 3. The American Academy of Pediatrics recommends offering parental reassurance, massaging the gums, using teething rings and using acetaminophen for 24 hours for the associated pain 4. However, the effects of teething on infant health have long been discussed and traditional beliefs regarding the topic are still not fully supported by scientific findings 5. Internet use is now part of the daily routine of nearly 3 billion people worldwide 6. The Internet is one of the largest medical libraries in the world 7.

Infant teething can be an irritating, stressful time for parents, especially new parents 8. Parents and caregivers are increasingly searching the Internet to gather information about their children’s health care 8-9.

The expansion of the Internet provides easier access to health and medical information than ever before. At least 95 million people have searched at least 1 of 16 major health topics online 10. This is problematic because little of the information found on the Internet is discussed with a medical practitioner; instead, it is used as the sole basis for decision-making 11. In addition, information acquired from the Internet might make patients less willing to adhere to their doctor’s advice, resulting in poor health outcomes 12.

In an effort to promote the quality and reliability of health information posted on the Internet, the Health on the Net Foundation developed a code of ethics (HONcode) for the certification of health-related websites 8,13. The HONcode outlines principles for disseminating quality, objective and transparent medical information tailored to readers 13. When accessing a website, four criteria are used to ensure the quality and accuracy of the content: author identity, financial support, content quality and privacy policy 8,12.

Parents typically either ask pediatricians or search the Internet to find information about child health problems and specific treatments. This study compared infant teething information gathered from publically employed pediatricians in Istanbul with that found on different Turkish websites (parenting, health, professional, news and commercial).

## METHODS

This study consisted of two parts.

### Part one

The first part of the study had a descriptive design. In May 2015, searches were conducted to identify websites with teething-related information using the Google search engine with different combinations of the Turkish equivalents of the following keywords: “teething”, “teething symptoms” and “infant teething”. To be included in the study, a website had to satisfy the following criteria: (a) be located in Turkey, (b) be written in Turkish and (c) provide parenting and child health information for consumers. Only websites having an Alexa traffic ranking of 100,000 or less were included in the study according to Kozuçh et al. (2015) 8,14. We initially found 78 parenting, health and news websites related to “teething”. After eliminating duplicate sites, portable document links, teething information presented in a question-and-answer format and blogs, 62 websites met the study inclusion criteria.

#### Data collection for the first part of the study

Two checklists were used to assess the quality and comprehensiveness of the teething-specific content on the 62 parenting and health websites according to Kozuch et al. 8. The first checklist focused on the credibility, relevance, author credentials, references and comprehensiveness of the teething content; the date of the last site or page update; and the presence of the HONcode or Utilisation Review Accreditation Commission (URAC) accreditation seal on each site. The HONcode tool bar 13 and URAC search directory were used to identify a site’s current approval status 8.

The second checklist included the categories of specific teething content (e.g., definition, clinical features), symptoms (e.g., drooling, loss of appetite) and management strategies (e.g., gingival massage, medicaments) reproduced from Schmitt (2011) and other studies 1-5,8,15.

### Part two

The second part was a cross-sectional study of 75 pediatricians at public hospitals who completed a structured self-administered questionnaire on information regarding symptoms attributed to teething and management strategies. This questionnaire was translated and modified from Wake and Hesketh (2002) 15. Written informed consent was obtained from the pediatricians participating in this part of the study and the study design was approved by the Ethics Committee of the School of Dentistry, University of Marmara (approval number: B.50.3.MAR.0.01.02/AEK/172).

### Data analysis

Data from the websites and questionnaires were entered into a Microsoft Excel spreadsheet and analyzed with descriptive statistics and chi-square tests using SPSS v17.0 (2015).

## RESULTS

### Results of part one

None of the websites were accredited by the HONcode regarding their compliance with ethical standards for credible, relevant and current health information on the Internet. Of the 62 included websites, the home page was last updated for 42 sites in 2015, for 4 sites in 2014, for 1 site in 2012 and for 1 site in 2010; 14 sites had no date listed. Of the 62 websites, 30 (48.4%) were parenting websites, 13 (21%) were professional websites, 9 (14.5%) were news websites, 6 (9.7%) were commercial websites and 4 (6.5%) were health websites. Nineteen of the websites provided the names of the authors of the teething-specific articles. Only three of the websites included a list of references to specific teething articles. Twenty-five (40.3%) websites indicated the date that their teething-related content was last updated, ranging which ranged from 2003 to 2015.

Table 1 summarizes the teething-specific content from the websites according to website type (Table 1). A total of 54 websites (87.1%) described infant teething as a normal developmental process. While 8 websites did not provide any information concerning the timing of infant teething, 87.1% of the 62 sites properly defined primary dentition as beginning between 4 and 6 months of age and ending at 2.5–3 years (Table 1).

Only two websites (one herbal health site and one commercial site) did not list any clinical features of teething, whereas 96.8% of the websites included lists of signs and symptoms thought to be associated with teething. Forty-nine (79%) websites discussed the probability that nonspecific clinical features (e.g., loss of appetite, diarrhea, crying, cough and fever) might also be associated with an undiagnosed illness. Thirty-five (56.5%) websites argued the importance of parental support during this potentially stressful time; there was a significant difference in mentioning parental stress among the different website types (*p*=0.057) (Table 1).

Fifty-two (83.9%) websites provided recommendations on non-pharmacological or pharmacological approaches to teething, including warnings or complementary strategies for infant teething (Table 1).

Of the 62 websites, 5 did not include warnings concerning teething symptoms that might worsen or extend for a period of time and 57 (91.9%) advised parents to call their primary care provider if they were unsure whether their infant was teething or had an illness, or for a recommendation regarding medication (Table 1).

The lists that were provided on the websites identified the most frequent signs of infant teething as drooling/perioral rash (91.9%), sleep disturbance (90.3%), inflamed/swollen/painful gums (88.7%), the need to chew or bite (87.1%), wakefulness (87.1%) and loss of appetite (75.8%). Other symptoms included diarrhea (51.6%), earaches or ear pulling (48.4%), persistent crying (40.3%), hematoma (32.3%), vomiting (21%) and fever >38.5°C (8.1%). The professional sites mentioned hematoma as a symptom of teething significantly (*p*=0.015) more often than did the other website types (Table 2). All websites identified fever as a sign of teething.

The most frequent management strategies given for infant teething were chewing non-chilled objects (82.3%), chewing chilled objects (80.7%), gingival massage (80.7%), paracetamol (45,2%), ibuprofen (21%), oral benzocaine (38.7%) and herbal remedies (8.1%). Of the included websites, 79% had warnings regarding medication use. The parenting, health and professional websites advised the use of teething gels significantly (*p*=0.035) less often than the commercial and news websites (Table 3). Other management strategies included keeping the face dry, applying a moisturizer to reduce the rash that can accompany infant teething and cuddling the child more often (27.4%).

### Results of part two

The average age of the pediatricians was 30.0±3.14 years and their tenure ranged from 1–14 years. All of the pediatricians were public employees. Toddlers were the main patients seen by 15 pediatricians (20%), while preschool children were the main patients seen by 40 pediatricians (53.3%) and schoolchildren were the main patients seen by 20 pediatricians (26.6%). Infant teething caused trouble always, sometimes or never according to 23 (30.7%), 21 (28%) and 31 (41.3%) of the pediatricians, respectively.

**Figure 1:**
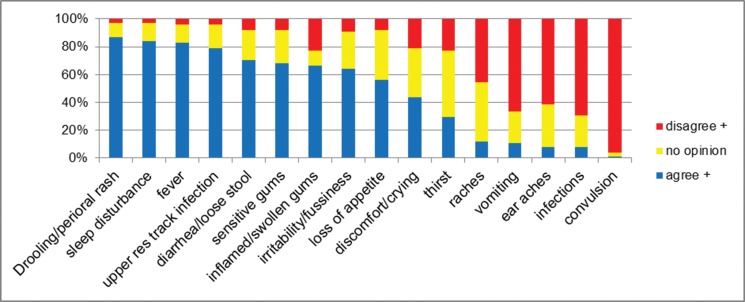
The most prevalent symptoms and signs believed to be associated with teething among pediatricians.

**Figure 2:**
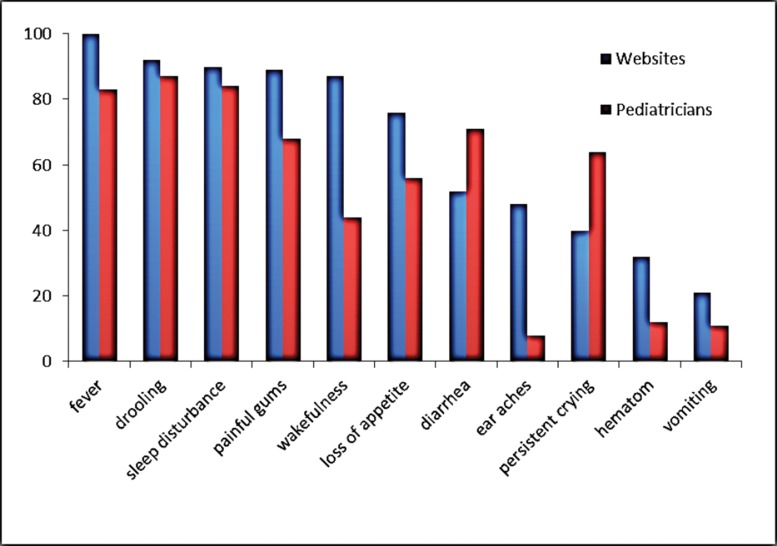
The most prevalent symptoms and signs believed to be associated with teething among pediatricians and the Internet.

Hypersalivation/drooling (86%), changes in sleep patterns (84%), fever (83%), chest infection symptoms (79%), diarrhea (71%), swollen or tender gums (68%), anxiety (64%) and loss of appetite (56%) were the most prevalent symptoms and signs believed to be associated with teething among the pediatricians. Earache (8%), infections (8%) and convulsions (1.3%) were among the least frequently associated symptoms (Figure 1). The symptoms found on the Internet and stated by the pediatricians are also shown in Figure 2. The pediatrician-recommended treatments for teething problems were teething rings (40%), oral benzocaine (24%) and paracetamol or ibuprofen (5.5%), although 23 of the pediatricians (30.7%) recommended nothing.

## DISCUSSION

The main findings of this study are as follows: 1) parenting websites provided more information about infant teething than other website types, 2) medications were not widely recommended by professional websites or pediatricians and 3) none of the included websites had a HONcode quality seal.

This study evaluated the differences found in teething information given on the Internet and by pediatricians in Turkey. Therefore, we searched only Turkish websites. The increasing availability of online health information provides opportunities to improve patient knowledge, but effective use of these resources depends on online health literacy and the quality and type of each website 16. Our Internet search showed that health information, including teething information, can be obtained from various website types. Our study found that parenting websites provided more information about infant teething than other types of websites. Kozuch et al. (2015) also searched parenting websites for the same purpose and found 16 parenting websites that included infant teething information in December 2012 8. In May 2015, we found 62 websites that contained information about infant teething; 30 of them were parenting websites, showing that parenting websites in Turkey are plentiful. However, as a website is easy to set up, there are potentially many biased and misleading electronic information sources that may not properly inform patients or parents 17. It is important that parents check for the HONcode accreditation quality seal as well as the qualifications and the background of a website’s author/sponsor. In this study, none of the websites had HONcode accreditation. Similarly, Kozuch et al. 8 found that only 3 of 16 websites had HONcode accreditation. A trustworthy website should list the authors of any included documents 12. In our study, all of the professional and most of the parenting websites listed author credentials. Similarly, Kozuch et al. (2015) found that most websites listed author credentials 8.

Infant teething is a cause of parental distress 8. Extensive information about infant teething was present on most of the websites included in the study. Parental stress was mentioned on parenting websites more often than on the other website types. Thirty-five (56.4%) of the websites argued the importance of parental support during this potentially stressful time and there was a significant difference in mentioning parental stress among the different website types (*p*=0.057). Kozuch et al. (2015) found that parental stress was mentioned on very few parenting websites 8. The nonspecific clinical features of infant teething commonly mentioned on all websites were similar to those described in the literature 1,4,12–14. The professional sites mentioned hematoma as a symptom of teething significantly (*p*=0.015) more often than did the other website types. Additionally, it appeared to be thought that severe systemic symptoms in infants are related to other diseases 2. In one study, bacterial infections were found in 7.1% of infants examined by a pediatrician 2. Consequently, the danger of attributing all symptoms to infant teething without ruling out other possible causes must be highlighted.

It is important to discuss the risks associated with using oral and topical medications, especially oral analgesics. In this study, medications were not widely recommended by professional websites or pediatricians, while paracetamol and teething gels were widely recommended by news and commercial websites. Trajanovska et al. found that parents reported rarely seeking the recommendation of a health professional about using over-the-counter medications to relieve infant teething symptoms 18. Kozuch et al. found that the majority of parenting websites recommended the use of acetaminophen, ibuprofen or teething gels for pain relief. In our study, the majority of websites provided information about medication warnings or possible adverse effects of administering medications for infant teething 8. Conversely, Kozuch et al. (2015) found that few parenting websites warned parents about the safe use of over-the-counter medications 8.

In another study, parents said that they would welcome more signposting towards useful online resources by healthcare professionals 19.

Previous studies have suggested that online information-seeking is generally viewed as augmenting professional advice, rather than replacing it 20-22. D’Auria provides helpful tips for health professionals to share with parents to help them identify trustworthy health information websites 23.

Although a website does not replace the advice of a health professional, information from a high-quality website can enrich educational and counseling efforts about child development. Nevertheless, parents should be informed by a health professional, especially for specific treatment strategies.

## AUTHOR CONTRIBUTIONS

Haznedaroglu E and Mentes A conceived the study idea and participated in its design and coordination. Haznedaroglu E performed the data collection for the whole study and drafted the manuscript. Mentes A performed the data analysis and interpretation and helped drafting the manuscript. Both authors read and approved the final version of the manuscript.

## Figures and Tables

**Table 1:** Teething-specific content on the websites according to their types.

	Parenting	Health	Websites Professional	News	Commercial	Total	*p*
Definition	28	3	11	6	6	54	0.35
Epidemiology	27	2	12	8	5	54	0.88
Clinical features	30	3	13	9	5	60	0.23
Unrecognized illness**	25	3	11	6	4	49	0.27
Parental stress/support	22	1	4	6	2	35	0.057*
Management strategies	25	2	11	9	5	52	0.46
Call primary care providers	28	2	13	8	6	57	0.62

* *p*≤0.05

** e.g., undiagnosed febrile illness, undiagnosed diarrhea

**Table 2:** The most frequent signs of infant teething according to website type.

	Parenting	Health	Websites Professional	News	Commercial	Total	*p*
Drooling/perioral rash	27	4	12	8	6	57	0.62
Inflamed/swollen/painful gums	26	4	11	8	6	55	0.55
Sleep disturbances	27	4	12	8	5	56	0.73
Need to chew or bite	26	4	13	8	3	54	0.23
Loss of appetite	20	4	11	8	4	47	0.34
Wakefulness	25	4	11	8	6	54	0.36
Diarrhea	14	4	7	6	1	32	0.75
Persistent crying	9	3	6	6	1	25	0.43
Vomiting	5	0	2	5	1	13	0.19
Fever >38.5°C	2	0	1	2	0	5	0.63
Earaches/pulling ears	12	2	7	7	2	30	0.30
Hematoma	4	2	8	3	3	20	0.015*

* *p*≤0.05

**Table 3:** The most frequent management strategies for infant teething according to websites’ type.

	Parenting	Health	Websites Professional	News	Commercial	Total	*p*
Gingival massage	24	4	10	8	4	50	0.76
Chewing chilled objects	25	2	9	9	5	50	0.70
Chewing unchilled objects	27	2	8	9	5	51	0.70
Paracetamol	12	1	5	5	5	28	0.09
Ibuprofen	6	0	2	4	1	13	0.51
Oral benzocaine	9	1	4	6	4	24	0.035*
Medication warnings	23	2	11	8	5	49	0.38
Herbal remedies	2	1	1	0	1	5	0.88
Other	10	1	1	2	3	17	0.81

* *p*≤0.05
